# Probing the linguistic cerebellum: a qualitative review of the effects of cerebellar neurostimulation on language processing

**DOI:** 10.3389/fpsyt.2026.1850241

**Published:** 2026-07-14

**Authors:** Sabrina Turker, Gesa Hartwigsen

**Affiliations:** 1 Brain and Language Lab, Department of Behavioral and Cognitive Biology, University of Vienna, Vienna, Austria; 2 Research Group Cognition and Plasticity, Max Planck Institute for Human Cognitive and Brain Sciences Leipzig, Leipzig, Germany; 3 Cognitive and Biological Psychology, Wilhelm Wundt Institute for Psychology, Leipzig, Leipzig University, Leipzig, Germany

**Keywords:** cerebellum, language, neurostimulation, non-invasive brain stimulation (NIBS), reading, review, tDCS, TMS

## Abstract

The cerebellum has historically been underrepresented in language research. However, converging evidence from lesion and neuroimaging studies supports its crucial involvement in phonological and semantic aspects of language processing, highlighting the relevance of cerebro-cerebellar circuits in language-related functions. Likewise, cerebellar neurostimulation has emerged as a promising tool for modulating sensorimotor and higher-order cognitive processes. A systematic search was conducted in PubMed and Web of Science following PRISMA guidelines and the present review synthesizes findings from 30 neurostimulation studies probing the causal role of the cerebellum for language processing. It summarizes transcranial electric and magnetic stimulation studies in neurotypical speakers and readers, and individuals with higher-order language disorders. Special emphasis is placed on studies combining cerebellar stimulation with behavioral interventions to assess the potential of improving language-related outcomes in clinical populations. Collectively, these studies support the role of the right posterolateral cerebellum as a key modulator of language functions, especially meaning-related operations.

## 1 Introduction

The cerebellum contains about four times more neurons than the cerebral cortex and a total of 80% of the brain’s neurons (1). Nonetheless, it has only gained little attention in cognitive neuroscience studies as compared to its larger, less wired counterpart (2, 3). While it does not possess specific cyto- or myelo-architectonic signatures like cortical brain regions (2, 4), it contributes to a wide array of human functions that are topographically arranged and spread across three lobes, which are separated by fissures into ten lobules: these are the anterior lobe (lobules I to V), the posterior lobe (lobules VI to IX) and the flocculonodular lobe (lobule X) (1, 4, 5).

The cerebellum interacts with cortical brain regions structurally and functionally. Three pairs of white matter tracts connect the cerebellum with the brainstem (cerebellar peduncles) (see summary in 1, 6). Histological findings in macaques provided first evidence for strong structural connectivity between communication-sensitive regions in the cortex and the cerebellum (7). A recent study using data from the Human Connectome Project aimed to map structural pathways between language-relevant cerebellar and cortical regions. They found that the volume of the language-specific cerebello-thalamo-cortical pathways was greater than the cortico-ponto-cerebellar pathway. Additionally, frontal lobe areas like the inferior frontal gyrus captured most projections from the cerebellum, with evidence for temporo-parietal regions (angular gyrus, superior temporal gyrus and temporal pole) showing higher connectivity relative to other temporal regions (8). Resting-state functional connectivity studies further demonstrate that posterolateral cerebellar regions, particularly crus I/II are intrinsically coupled to fronto-parietal, default-mode, and language-related cortical networks (9, 10).

Functionally, the cerebellum is connected to the cortex via reciprocal connections, called cortico-cerebellar loops (4, 11). These loops connect the posterior cerebellum (lobules VI, VII) with prefrontal, posterior parietal, temporal and cingulate cortices, making the cerebellum crucial for higher-order cognitive functions. Together, these structural and functional connectivity patterns provide a plausible anatomical substrate through which cerebellar regions may influence language processing, not via isolated local computations, but through coordinated interactions with distributed cortical language and executive-control networks.

Traditional accounts have posited a right-lateralized organization of language in the cerebellum based on lesion studies revealing that primarily damage to right posterior cerebellar regions leads to language impairments. These findings from lesion studies have informed several influential reviews discussing cerebellar contributions to language processing and the integration of the cerebellum into linguistic models (e.g., 12–14). However, recent meta-analytic evidence speaks in favor of a critical functional role of both cerebellar hemispheres for language and reading (15, 16).

In terms of specific linguistic operations supported by the cerebellum, specifically semantic (i.e., meaning-related) and phonological (i.e., sound-related) tasks lead to activation in bilateral crus I and lobules VI and V, with slight differences in location of lobule VI between phonology and semantics (15). More specifically, the authors showed that primarily left and right cerebellar lobules VI were involved in semantic and phonological processes. However, phonological tasks yielded higher activation and larger and more consistent activation was found in the right cerebellum. Right cerebellar lobule VI was also linked to visual language processing and speech production, whereas crus I/II were more engaged during overt speech production. A potential role of both cerebellar hemispheres for language is also supported by a recent clinical study with people with aphasia, which revealed that language impairments occur after both right and left cerebellar damage (17). This is in line with a previous review and meta-analysis that reported stable cerebellar language deficit subtypes following cerebellar brain damage, which matched symptoms of cerebral cortex lesions: for instance, one cluster of cerebellar symptoms resembled Broca’s aphasia, with patients experiencing primarily expressive agrammatism and difficulties in grammar comprehension (18). This supports the notion that a loss of the excitatory input from the cerebellum to cortical language areas may cause language symptoms matching those of lesions in the specific cortical area (18, 19).

In the past three decades, non-invasive brain stimulation (NIBS) methods have become valuable tools to investigate human cognition (20, 21). NIBS is usually applied either offline (i.e., before a task) or online (i.e., during a task), depending on the research question. The most popular methods are transcranial magnetic stimulation (TMS) and transcranial electrical stimulation (tES), with new methods still emerging and gaining popularity, including transcranial ultrasound stimulation (tUS) (22) or temporal interference (TI) stimulation (23). As the included studies applied either TMS or a form of tES, these two methods will be discussed in more depth in the following paragraph.

A single TMS pulse influences cortical neurons via a brief, strong magnetic field that induces a short-lived electric current in the brain, which can temporarily excite or inhibit neural activity (24). Most physiological TMS research has focused on the motor cortex because its effects can be directly measured through motor evoked potentials, which reflect corticospinal excitability and can even produce observable muscle movements. In non-motor brain regions, TMS effects are harder to measure and are typically assessed through changes in behavior or neural activity during cognitive tasks, such as language processing (for more details, please refer to 24 and 25). TMS protocols include single pulses, paired pulses and short or longer bursts of repetitive TMS (rTMS) at different frequencies.

In contrast, transcranial direct current stimulation (tDCS), the most applied form of tES, modulates cortical excitability via weak direct electrical currents applied to the scalp through surface electrodes for longer durations (usually between 10 and 30 minutes). tDCS produces polarity-dependent changes in neuronal activity through shifts in resting membrane potential (26, 27). Anodal stimulation typically increases excitability, while cathodal stimulation decreases it, with immediate effects driven by membrane depolarization or hyperpolarization and longer-lasting after-effects likely involving synaptic plasticity mechanisms such as long-term potentiation or depression. These effects can persist beyond the stimulation period and are commonly measured using behavioral performance, neurophysiological markers, or neuroimaging. More recent electrical stimulation techniques aim to modulate cortical rhythms via alternating currents at different frequencies (transcranial alternating current stimulation) or varying frequencies (transcranial random noise stimulation) (28).

NIBS studies have already provided crucial insight into the contribution of various brain regions to language processing (29, 30). Existing studies have explored the causal contribution of specific brain regions for language processing by either (a) disrupting its functioning, or (b) ‘enhancing’ its functioning especially in clinical conditions (e.g., in people with brain disorders such as aphasia). A recent meta-analysis by Klaus and Schutter (31) investigated the effects of NIBS on language production in healthy speakers, providing evidence for the effectiveness of both TMS and tDCS on picture naming and verbal fluency. Another meta-analysis explored the effects of inhibitory NIBS to homologous language regions in post-stroke aphasia, revealing a moderate effect size for improved naming accuracy after inhibitory rTMS and cathodal tDCS (32). The efficacy of NIBS in post-stroke aphasia was also recently analyzed in an extensive meta-analysis (33). The authors reported stronger effects of tDCS than low-frequency rTMS, albeit the latter is the most applied method in post-stroke aphasia recovery (see also 34). Notably, the effects depend on the chosen target, based on the most severely affected domain, and other internal (e.g., first language, age) and external (e.g., therapy period and intensity) factors (33). Overall, the effect sizes of therapeutic NIBS studies for aphasia therapy are moderate and numerous questions remain open, including the optimal stimulation protocol and potential changes of stimulation sites and protocols across the course of aphasia recovery (e.g., 35). Moreover, most of the existing studies include small sample sizes and targeted exclusively cortical regions, with cerebellar stimulation being only recently explored for aphasia therapy (e.g., 36, 37).

Although previous consensus and review papers have addressed cerebellar NIBS across motor, cognitive, and clinical domains, language-specific investigations have typically only been discussed as part of broader overviews (38–41). However, language processing cannot be equated with general cognitive processing, which complicates the interpretation of existing findings with respect to the specific causal contribution of the cerebellum to language functions. Consequently, a systematic synthesis of cerebellar neurostimulation studies targeting core linguistic domains, including semantic or sentence-level processing, as well as speech production, and language rehabilitation is still lacking. Especially given the role of the cerebellum for reading and related disorders (16, 42–44), the integration of NIBS studies using reading tasks is necessary.

The present review provides an in-depth synthesis of previous NIBS studies targeting the cerebellum and attempts a domain-specific integration of behavioral and neuroimaging evidence to clarify the functional contribution of the cerebellum to language processing.

## 2 Methods

In the present review, we aimed to systematically evaluate the causal effects of cerebellar NIBS on language processing and reading across healthy individuals and clinical populations, with a particular focus on domain-specific behavioral and neurophysiological outcomes. To that end, we conducted a systematic review following the PRISMA guidelines (45, 46). The research question was defined according to the PICOS framework. Specifically, we included studies investigating healthy individuals and clinical populations with language impairments (population), in which NIBS was applied over the cerebellum (intervention), compared to sham stimulation or control conditions where applicable (comparator). Studies were required to report behavioral and/or neurophysiological outcomes related to language or reading (outcomes) and to employ experimental study designs, including cross-sectional or longitudinal approaches (study design).

The systematic literature search was conducted in PubMed and Web of Science using combinations of keywords related to cerebellar stimulation and language processing between December and March 2026 (last search on March 8, 2026).

The following search strategies were applied:

PubMed:

- (“cerebellum” AND “TMS”)
- (“cerebellum” AND “stimulation” AND “language”)
- (“cerebellum” AND “stimulation” AND “reading”)

Web of Science:

- (“cerebellum” AND “neurostimulation” AND “language”)
- (“cerebellum” AND “neurostimulation” AND “reading”)
- (“cerebellum” AND “stimulation” AND “language”)
- (“cerebellum” AND “stimulation” AND “reading”)

**Figure 1:**
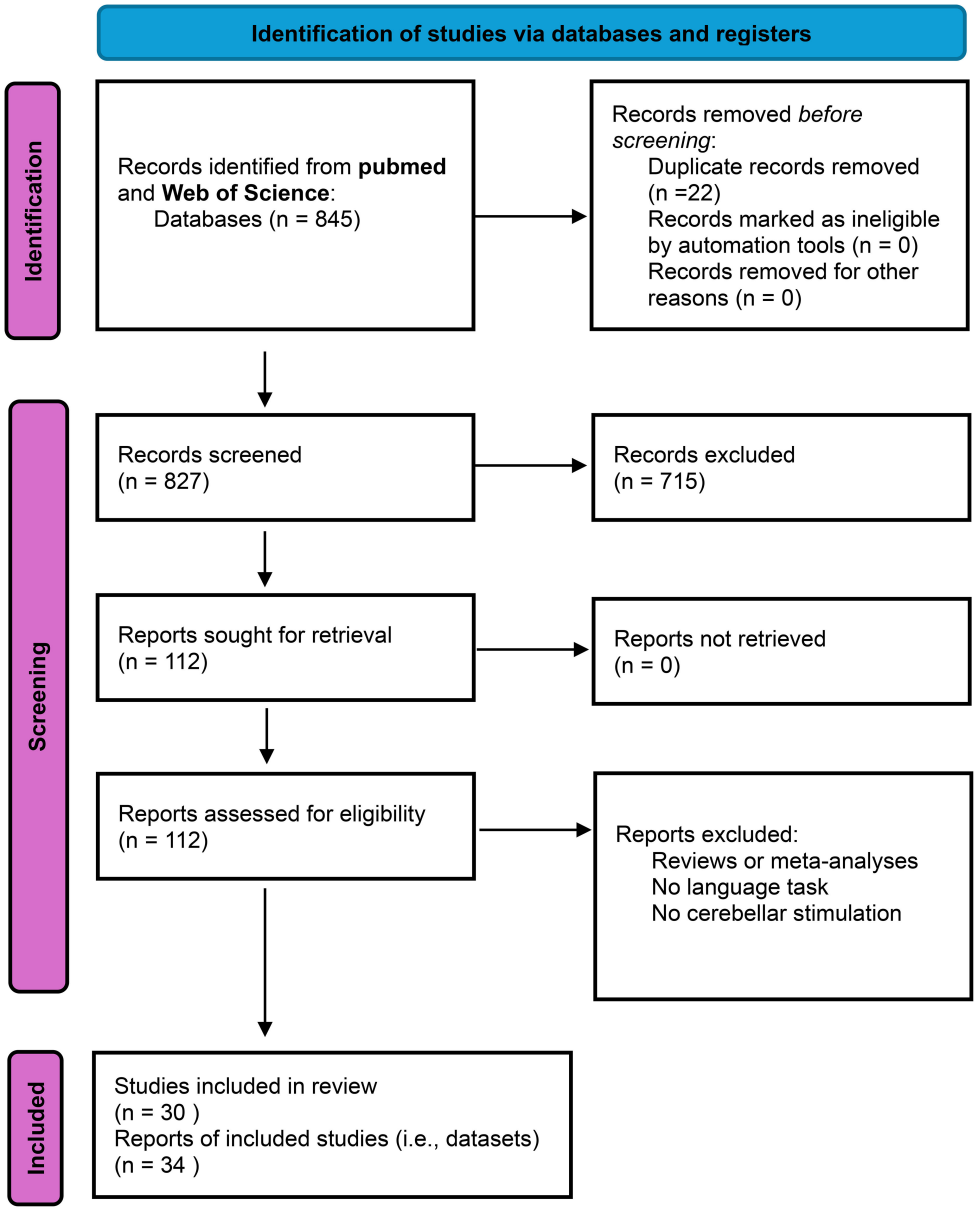
Flow chart of study identification, screening and selection process for the present review.

Search terms were entered as free-text keywords. Additional synonyms were explored but yielded no additional relevant studies. Given the relatively small and highly specialized literature on cerebellar neurostimulation and language processing, broader search combinations produced a large number of irrelevant records without identifying additional eligible studies. The final search strategy was therefore intentionally focused to maximize specificity while maintaining coverage of the relevant literature. Reference lists of relevant reviews and included studies were additionally screened to identify potentially eligible articles. The review followed PRISMA guidelines and predefined inclusion and exclusion criteria; however, the review protocol was not formally preregistered. No restrictions were applied regarding publication date. Searches were limited to studies published in English. The search yielded 845 records (PubMed: 591; Web of Science: 254) and were screened (title and abstract). Following screening and eligibility assessment, 30 studies were included in the review. Of these, one included three experiments and two included two experiments, resulting in a total of 34 included experiments (see **Figure 1**). Titles and abstracts were screened by the first author and verified by the last author. Please note that the review was not registered and no protocol was prepared.

Inclusion criteria were the following:

- Cross-sectional or longitudinal study in which one or more NIBS methods (e.g., TMS, tES, TUS) were applied over a region in the human cerebellum.
- Administration of a speech or language task before and after or during stimulation.
- Assessment of stimulation effects in terms of behavior or neural changes through secondary methods (functional magnetic resonance imaging (fMRI), functional near-infrared spectroscopy (fNIRS), electroencephalography (EEG), magnetoencephalography (MEG)).
- Studies combining cerebellar neurostimulation with additional neurophysiological or neuroimaging methods (e.g., fMRI, EEG, fNIRS, MEG) were included, provided that the primary intervention involved NIBS of the cerebellum and that language- or reading-related behavioral and/or neural outcomes were reported.

Exclusion criteria were the following:

- Single case studies
- Study protocols

The following information was extracted from each included study using a standardized data extraction form in accordance with PRISMA guidelines: study identification (first author, DOI); stimulation protocols (type of NIBS, including TMS, tES variants such as tDCS); stimulation parameters (online vs. offline application, intensity); and the use of additional methods (e.g., multimodal approaches). Information regarding the experimental setup was also collected, such as the presence and characteristics of speech-language therapy (SLT) components, the targeted cognitive or linguistic function, and the anatomical stimulation target (reported either as MNI coordinates or descriptive localization), including specification of cerebellar lobules or crus regions where available. Sample characteristics were extracted, including population type (healthy vs. clinical) and sample size per group. Study design features were recorded, such as experimental design (e.g., within- or between-subjects), presence of longitudinal assessments, and timing of outcome measures. Task-related information included the type of language or speech task employed and detailed task descriptions. Finally, main outcomes and findings were extracted, focusing on behavioral and/or neurophysiological effects associated with cerebellar stimulation.

A qualitative synthesis was conducted. Studies were grouped according to stimulation method, task domain, and population. A shorter version of this table with all relevant information can be found in **Table 1**. The full table with explicit task descriptions and full result sections is available via the OSF project page: 10.17605/OSF.IO/9C4PS. Given the heterogeneity of study designs and outcome measures, a formal risk of bias assessment was not conducted. We note that a formal quantitative risk-of-bias assessment was not conducted due to substantial heterogeneity in stimulation modalities, study designs, outcome measures, and experimental paradigms. Instead, methodological quality was evaluated qualitatively during data synthesis, as this approach was considered more appropriate than applying a single standardized risk-of-bias instrument (e.g., ROBINS-I or Cochrane RoB tools) across all included studies. The structured qualitative evaluation is further discussed in Section 4.1. Comparable approaches have been adopted in previous systematic reviews of cerebellar neurostimulation and language-related NIBS research (38, 41, 74, 75).

**Table 1:** Summary of included studies with information on the sample, sample size (N), the chosen NIBS method, its combination with speech-language-therapy (SLT), NIBS timing, intensity and targets. Additionally, targeting techniques, main tasks, outcomes and key findings are provided.

Study	Sample	N	NIBS	SLT	Timing	Intensity	Region	Targeting	Task	Outcome	Key finding
Argyropoulos, 2011 (47)	Healthy	8	TMS (cTBS)		Offline	45% output	R cer (2 targets)	Manual	Semantics	Behavioral	Medial cerebellar cTBS reduced lexical decision accuracy, lateral cTBS did not
Argyropoulos & Muggleton, 2013 (48)	Healthy	13/13	TMS (cTBS)		Offline	45% output	R cer (2 targets)	Manual	Semantics	Behavioral	Lateral cerebellar cTBS increased semantic priming effects; medial cerebellar TBS did not
Bongaerts et al. 2022 (49)	Healthy	12/12/12	tDCS (a,s)		Online	2 mA (~5 min)	R cer	Manual	Semantics, Sentence processing,Fluency	Behavioral	No significant behavioral effects
Carthery-Goulart et al. 2026 (50)	Clinical (Aphasia)	6	HD-tDCS (a,s)		Online	2 mA (20 min)	R cer	Manual	Word naming	Behavioral	Sham tDCS improved verb naming compared to anodal tDCS
Coemans et al. 2025 (51)	Clinical (PPA/ Aphasia)	7	tDCS (a,s)	Yes	Online	2 mA (20 min)	R cer	Manual	Language battery	Behavioral	Anodal tDCS improved language recovery and inhibitory control
Dai et al. 2026 (52)	Clinical (Aphasia)	20/20/20	TMS (iTBS, cTBS)	Yes	Offline	80% rMT	R cer	Manual	Language battery, Cognition	Behavioral	cTBS improved language, cognition & quality of life; no effect of iTBS
Dave et al. 2020 (53)	Healthy	24	TMS (cTBS, rTMS)		Offline	80% rMT	R cer R occipital	MNI	Reading, Memory	Behavioral + EEG	cTBS improved episodic memory, rTMS did not; rTMS influenced neural signals for semantic prediction, cTBS did not
DeMarco et al. 2021 (54)	Clinical (Aphasia)	10/14	tDCS (a,s)	Yes	Online	2 mA (20 min)	R cer	Manual	Language battery	Behavioral	No improvement of language scores, but effects on picture description post-testing and at follow-up
D’Mello et al. 2017 (55)	Healthy	18/14	tDCS (a,s)		Online	1.5 mA (20 min)	R cer	Manual	Sentence processing	Behavioral+ fMRI	No behavioral effects; anodal tDCS increased activation within right lobule VI/crus I during predictive sentences and altered resting-state networks for language (crus I to left cuneus)
Gilligan & Rafal, 2019 (56)	Healthy	21/20	TMS (cTBS)		Offline	80% rMT	L + R cer	Manual	Semantics	Behavioral	Right cerebellar cTBS increased and left cerebellar cTBS decreased semantic priming effect
Gatti et al. 2020 (57) (Exp. 1)	Healthy	24	TMS (triple pulse)		Online	100% MT	R cer Vertex	MNI	Semantics	Behavioral	TMS decreased accuracy for related pairs compared to vertex; non-sign. trend for faster responses
Gatti et al. 2020 (57) (Exp. 2)	Healthy	20	TMS (triple pulse)		Online	100% MT	R cer R visual	MNI	Semantics	Behavioral	TMS decreased accuracy for related pairs compared to right visual cortex
Gatti et al. 2020 (57) (Exp. 3)	Healthy	21	TMS (triple pulse)		Online	100% MT	R cer Vertex	MNI	Letter recognition	Behavioral	No behavioral effects of TMS
Gatti et al. 2021 (38) (Exp. 1)	Healthy	24	TMS (triple pulse)		Online	100% MT	R cer Vertex	MNI	Semantics	Behavioral	TMS selectively worsened discriminability for critical lures, leading to higher likelihood of false memories
Gatti et al. 2021 (38) (Exp. 2)	Healthy	32	TMS (triple pulse)		Online	100% MT	R cer Vertex	MNI	Semantics	Behavioral	The higher the semantic association between new and studied words, the larger the effect of TMS (i.e., higher memory impairment)
Johnson et al. 2026 (36)	Clinical (Aphasia)	19	tDCS (a,c,s)	Yes	Online	2 mA (20 min)	R cer	Manual	Language battery	Behavioral + dMRI	Baseline white matter tract metrics (left fronto-parietal to right cerebellum) predicted language recovery
Kim et al. 2024 (58)	Clinical (Aphasia)	14	tDCS (a,c,s)	Yes	Online	2 mA (20 min)	R cer	Manual	Language battery	Behavioral	tDCS improved only functional communication but not general language assessment
Lametti et al. 2018 (59)	Healthy	30/30	tDCS (a,s)		Online	2 mA (16 min)	R cer L motor	Manual	Speech production	Behavioral	Anodal tDCS to motor cortex or cerebellum improved sensorimotor learning in speech with differential effects on formants
Lee et al. 2024 (60)	Healthy	25	tDCS (a,c,s)		Offline	2 mA (20 min)	R cer	Manual	Reading	Behavioral	Cathodal tDCS reduced single-word reading fluency, but the effect did not survive correction for multiple comparisons
Lesage et al. 2012 (61)	Healthy	22	rTMS		Online	55% output	R cer Vertex	Manual	Sentence processing	Behavioral + eye-tracking	TMS caused delayed eye fixations to target objects predicted by sentence content; no effect on eye fixations in sentences without predictable content
Marangolo et al. 2018 (62)	Clinical (Aphasia)	12	tDCS (c,s)	Yes	Online	2 mA (20 min)	R cer	Manual	Word generation	Behavioral	Cathodal tDCS improved verb generation
Miall et al. 2016 (63)	Healthy	73	tDCS (a,c,s)		Online	2 mA (20 min)	R cer	Manual	Sentence processing	Behavioral	Cathodal tDCS disrupted sentence comprehension, while anodal tDCS facilitated it
Petríková et al. 2023 (64)	Healthy	45/45/46	tDCS (a,c,s)		Online	2 mA (20 min)	R cer	Manual	Semantics, Sentence processing	Behavioral	Anodal tDCS facilitated retrieval of sequentially related concepts within free-associative word chains but had no influence on semantic control or switching
Rice et al. 2021 (65)	Healthy	18/14/11	tDCS (a,s)		Offline	1.5 mA (15 min)	R cer (2 targets)	Manual	Sentence processing	Behavioral + fMRI	Anodal posterolateral (VII) tDCS improved sentence comprehension and increased activation in left fronto-temporal cortices
Rowe et al. 2025 (66)	Healthy	35/35/35	HD-tDCS (a,s)		Online	2 mA (20 min)	R cer L pIFS	Manual	Speech production	Behavioral	Anodal HD-tDCS Improved syllable motor learning
Runnqvist et al. 2016 (67)	Healthy	16	rTMS		Offline	60% output	L cer R cer	Not reported	Speech production/reading	Behavioral	Right cerebellar TMS impaired language production compared to left cerebellar stimulation
Sebastian et al. 2020 (37)	Clinical (Aphasia)	24	tDCS (a,c,s)	Yes	Online	2 mA (20 min)	R cer	Manual	Word naming	Behavioral	Greater naming gains following both anodal and cathodal stimulation, but strongest effect for cathodal
Spielmann et al. 2017 (68)	Healthy	24	tDCS (c,s)		Offline	2 mA (20 min)	R cer	Manual	Reading, Generation	Behavioral	No immediate behavioral effect; delayed negative trend one week post-stimulation
Turkeltaub et al. 2016 (69) (Exp. 1)	Healthy	15/16	tDCS (a,c,s)		Offline	2 mA (20 min)	R cer (2 targets)	Manual	Verbal fluency	Behavioral	Only tDCS over right posterolateral cerebellum (lobule VII) improved phonemic fluency
Turkeltaub et al. 2016 (69) (Exp. 2)	Healthy	16/11	tDCS (a,s)		Offline	1.5 mA (15 min)	R cer	Manual		fMRI	Increased language network connectivity
Xu et al. 2025 (70)	Clinical (Aphasia)	8/6	tDCS (c,s)	Yes	Offline	2 mA (20 min)	R cer	Manual	Language battery	Behavioral + fNIRS	tDCS of lobule VII increased interaction between language regions and speech motor control areas
Yuan et al. 2023 (71)	Healthy	23/22/22	HD-tDCS (a,s)		Online	2 mA (14 min)	L cer R cer	Manual	Word naming	Behavioral	anodal tDCS over the right cerebellum optimizes language control performance in bilinguals; higher L2 proficiency showed better stimulation effect
Yuan et al. 2025 (72)	Healthy	30	tDCS (a,c,s)		Online	2 mA (16 min)	R cer L motor	Manual	Speech perception	Behavioral	No behavioral effects of tDCS
Zheng et al. 2025 (73)	Clinical (Aphasia)	22/25	tDCS (a,s)	Yes	Online	2 mA (20 min)	R cer	Manual	Language battery	Behavioral	No added benefit of tDCS beyond benefits of SLT

For studies with between-subject designs, all group sizes are reported. Moreover, information on additional interventions in the form of speech and language therapy (SLT), the stimulation (offline vs. online), stimulation details and the targets is provided. For all tDCS studies, information on whether stimulation of the cerebellar target was anodal (a), cathodal (c) or sham (s) is provided in brackets in the NIBS column. In cases where different locations within one cerebellar hemisphere were targeted, the number of targets is provided in brackets. Last, the table includes information on the location of the target, the main task, outcome and the main findings. EEG, electroencephalography; cer, cerebellum; min, minutes; fMRI, functional magnetic resonance imaging; fNIRS, functional near-infrared spectroscopy; dMRI, diffusion MRI; iTBS, intermittent theta burst stimulation; cTBS, continuous theta burst stimulation; PPA, primary progressive aphasia; SLT, Speech-language-therapy; a, anodal; c; cathodal; s, sham.

## 3 Results

### 3.1 General characteristics of NIBS studies

**Figure 2:**
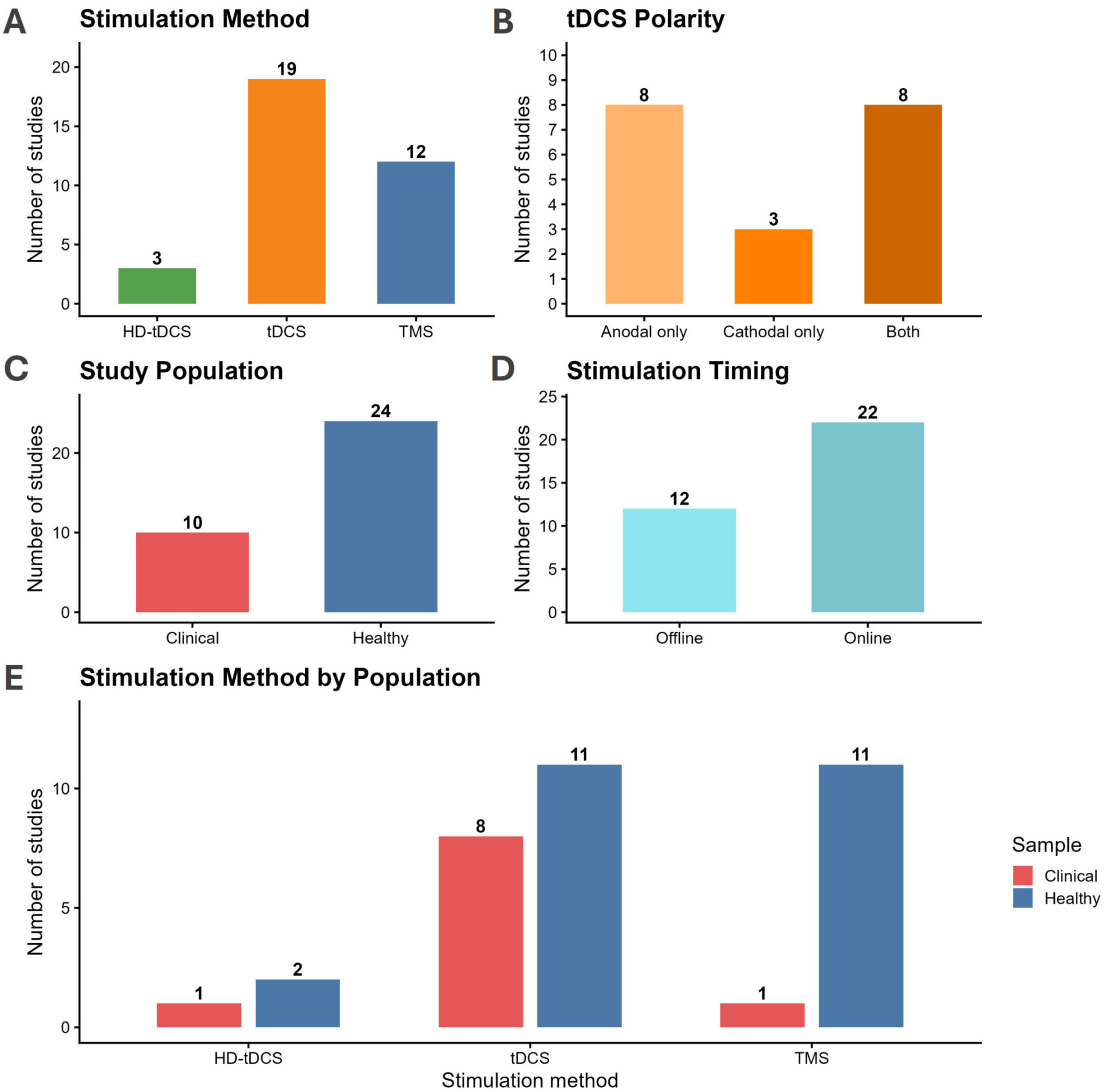
Descriptive summary of the included datasets. **(A)** The number of studies in which the different NIBS methods (HD-tDCS, tDCS, TMS) were used. **(B)** The tDCS polarity across the included tDCS studies (either just anodal, just cathodal or both in one experiment). **(C)** The number of studies investigating either healthy or clinical samples. **(D)** The stimulation timing across all included studies (i.e., either offline or online). **(E)** An illustration of the distribution of NIBS methods across study populations.

Thirty experimental studies (34 experiments) were included in the final dataset for the present review (cf. **Table 1**). **Figure 2** provides a visual summary showing the distribution of NIBS methods (HD-tDCS, tDCS, TMS) (A), tDCS polarity across tDCS studies (either just anodal, just cathodal or both in one experiment) (B), the specific sample (healthy vs. clinical) (C), stimulation timing (offline or online – i.e., prior to the task or during the task) (D) and the interaction of stimulation method with the study population (E).

TMS protocols include single pulses, paired pulses and short or longer bursts of repetitive TMS (rTMS) at different frequencies. Single pulses, paired-pulse protocols, or short TMS bursts can be used to probe the relevance of specific time windows when applied during a task, thereby providing insight into the temporal dynamics of specific processes. In contrast, longer rTMS bursts are typically used to cover extended stimulation periods during a task when the exact timing of the relevant process is unknown. Moreover, they can also induce longer lasting after-effects by modulating plasticity in the stimulated area or network. We note that most of the reviewed studies applied rTMS before the task to modulate cerebellar activity over a prolonged period. Six studies combined neurostimulation with neural measures, including task-based fMRI, resting-state fMRI, EEG, or fNIRS. The right cerebellar hemisphere was the primary target in all studies. Stimulation most frequently targeted posterolateral regions corresponding to crus I, crus II, and lobule VII as a more general target. Fewer studies examined anterior cerebellar regions (e.g., lobule V), medial–lateral subdivisions, or the left cerebellum (n = 3). Some studies included comparison sites such as the vertex, primary motor cortex, or occipital cortex, although many relied on sham-controlled designs.

Most investigations employed single-session designs (n = 23). Eight studies implemented repeated stimulation protocols across multiple days or weeks; all multi-session designs were conducted in clinical populations and were typically combined with structured SLT. Several of these studies included follow-up assessments to evaluate longer-term changes in performance.

**Figure 3:**
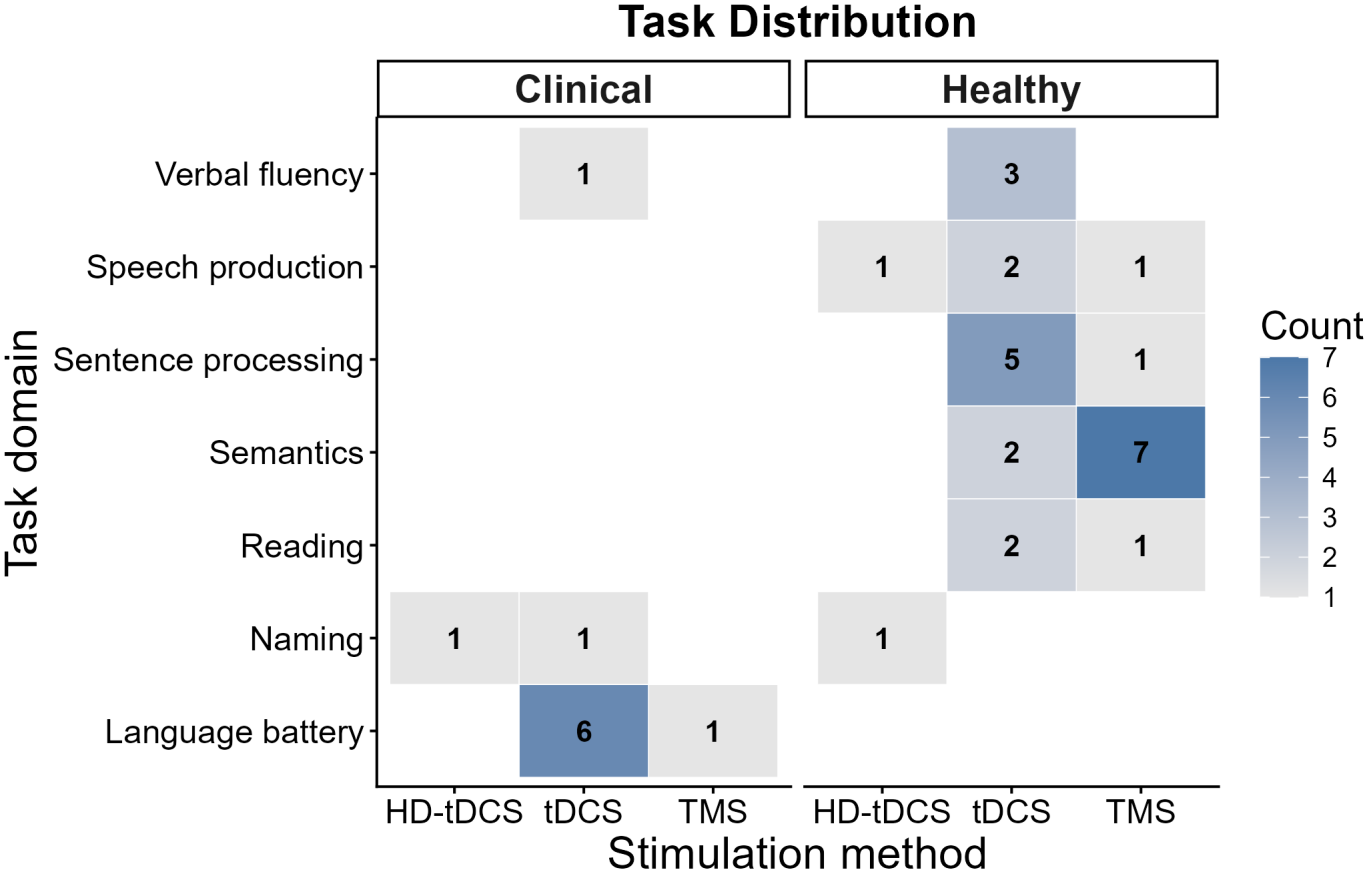
Distribution of task domains across study samples (healthy vs. clinical). Most studies with healthy participants employed some form of semantic tasks, while most studies with patients (aphasics) used large language assessment batteries to detect improvements due to NIBS.

For descriptive purposes, the included studies can be grouped into seven broad language-related domains: (1) semantics, (2) sentence processing, (3) speech production, (4) verbal fluency, (5) reading, (6) naming (and generation), and (7) language battery/language assessment (e.g., aphasia batteries). The distribution of employed tasks for the two study samples is shown in **Figure 3**.

For the simplicity of result interpretations, tasks will be further grouped into three main categories, namely (1) semantics (including all priming, predictive processing, lexical decision tasks), (2) speech production (including verbal fluency, generation) and (3) overall language or sentence processing (including large batteries, sentence comprehension and processing). The included reading studies tap into categories (1) and (2) and will be discussed separately.

### 3.2 Behavioral effects of cerebellar neurostimulation in healthy individuals

Across studies in healthy participants, cerebellar stimulation effects were functionally specific and regionally dissociable, with the most consistent findings observed for the right posterolateral cerebellum (crus I/II, lobules VI/VIIa).

The right posterolateral cerebellum appears to support semantic prediction and processing, with stimulation effects depending on both task demands and hemispheric location. In several studies, disruptive stimulation (rTMS/cTBS) over the right posterolateral cerebellum reliably impaired semantic processing, including reduced accuracy in semantic judgments, increased false memories, and diminished sensitivity to semantic associations. Importantly, one study demonstrated that these effects were modulated by semantic relatedness, with stronger impairments observed for highly associated word pairs, suggesting a role in context-dependent semantic integration. In contrast, stimulation of the right medial cerebellum selectively impaired lexical decision accuracy, indicating functional heterogeneity within the cerebellum. Complementing these disruptive effects, anodal tDCS facilitated the retrieval of semantically related concepts in associative chain tasks. Lateralized effects were further supported by findings showing that left cerebellar stimulation decreased, whereas right cerebellar stimulation increased semantic priming, pointing to hemispheric specialization within posterolateral cerebellar regions.

The included studies also suggest that the cerebellum contributes to predictive and context-driven sentence processing, with polarity-dependent effects indicating facilitatory versus disruptive modulation of these processes. Specifically, TMS over the right cerebellum disrupted anticipatory eye movements during sentence comprehension, but only in predictable contexts, highlighting a role in context-based prediction. Similarly, cathodal tDCS impaired sentence comprehension, whereas anodal stimulation of the posterolateral cerebellum improved performance.

With respect to speech production and reading, the right cerebellum appears to play a key role in speech motor adaptation and fluency, with effects extending to language control mechanisms. Anodal tDCS and HD-tDCS over the right cerebellum improved sensorimotor adaptation in speech, syllable motor learning, and phonemic fluency. In some cases, these effects were comparable to those observed following motor cortex stimulation, although cerebellar stimulation produced more specific adaptation patterns. TMS studies further showed that right cerebellar stimulation impaired speech production, whereas left cerebellar stimulation had no effect, again supporting right-lateralized contributions. Additionally, right cerebellar stimulation improved language control in bilinguals, particularly in individuals with higher second-language proficiency, suggesting an ability-dependent involvement of the cerebellum in specific linguistic operations.

Evidence for cerebellar involvement in reading was limited and less consistent compared to other language domains. Cathodal tDCS over the right cerebellum showed a tendency to reduce single-word reading fluency, although this effect did not survive correction for multiple comparisons. Other studies including tasks involving word and sentence reading did not report reliable behavioral modulation, suggesting that cerebellar contributions to reading may be weaker or more variable than those observed for semantic or speech production processes.

However, not all studies reported behavioral modulation. Several tDCS studies (anodal and cathodal) found no significant effects across language tasks. One TMS study reported no effects in a letter recognition task, suggesting that cerebellar contributions may be limited to higher-order linguistic processing rather than basic perceptual functions. Consistent with this interpretation, no reliable effects of cerebellar stimulation on speech perception were observed.

Finally, beyond the language domain, one study reported that cTBS over crus I improved episodic memory, suggesting partially distinct cerebellar contributions to broader cognitive and domain-general processes.

### 3.3 Behavioral effects of cerebellar neurostimulation in clinical populations

In contrast to studies in healthy participants, clinical studies primarily investigated the effects of repeated cerebellar stimulation protocols, most often in combination with SLT and, in some cases, including longitudinal study designs. Although findings were more variable, evidence suggests a potential positive effect of cerebellar neurostimulation in post-stroke aphasia and primary progressive aphasia. However, stimulation effects appear to depend on therapy context, stimulation parameters, and individual patient characteristics.

Most clinical studies focused on language recovery, particularly naming performance, language battery scores, and functional communication measures. Several studies reported beneficial effects of cerebellar stimulation on language recovery, particularly when combined with SLT. For example, repeated tDCS over the right posterolateral cerebellum enhanced language recovery and inhibitory control in bilingual aphasia, and improvements in naming performance were observed following multi-session stimulation protocols. Similarly, stimulation was associated with gains in functional communication, particularly in qualitative aspects of communication rather than global language independence.

However, effects were not uniformly observed across studies or outcome measures. Some studies reported improvements restricted to specific tasks (e.g., verb generation or untrained naming items), while others found no additional benefit of cerebellar stimulation beyond the effects of SLT alone. In one study, stimulation improved quality of life measures without significantly affecting core language scores.

With respect to stimulation parameters, both anodal and cathodal tDCS were associated with behavioral improvements, and in some cases, cathodal stimulation produced stronger effects on naming performance. Similarly, TMS studies suggested that cTBS protocols may be more effective than iTBS, although evidence remains limited.

Multi-session designs, typically conducted over several weeks, were associated with sustained improvements, including effects maintained at follow-up assessments. However, the frequent combination with SLT makes it difficult to disentangle the specific contribution of cerebellar stimulation from general therapy effects.

Taken together, cerebellar stimulation may support specific aspects of language recovery, but effects are task-dependent and not consistently generalizable. Evidence suggests that cerebellar stimulation may act as a modulatory or facilitatory tool, enhancing therapy-induced plasticity rather than directly driving behavioral improvements. Additionally, recovery patterns do not consistently follow classical cortical excitation–inhibition models, as stimulation effects did not adhere to a simple polarity-dependent rule.

Finally, several studies reported no additional improvement beyond SLT, particularly in standardized language assessments, while others observed improvements only after statistical adjustment or in secondary outcome measures. This variability suggests that stimulation effects are context-dependent and likely influenced by patient characteristics, intervention protocols, and outcome measures.

### 3.4 Neurophysiological effects of cerebellar neurostimulation

Six studies combined cerebellar stimulation with neural measures, including task-based fMRI, resting-state fMRI, EEG, and fNIRS (see **Table 1**). These studies extend behavioral findings by examining how cerebellar stimulation influences activity and connectivity within distributed language networks, sometimes even in the absence of behavioral changes.

A small number of studies assessed task-related activation changes following tDCS over the right posterolateral cerebellum (primarily lobule VII/crus I). In sentence completion paradigms, anodal stimulation was associated with increased activation within right cerebellar regions during predictive sentence processing. Importantly, stimulation also modulated activity in cortical language areas, including left inferior frontal and temporal regions. In some cases, behavioral performance showed modest or no change, despite measurable alterations in neural activation. Another study directly contrasted stimulation of posterior (lobule VII) and anterior (lobule V) cerebellar regions. Behavioral improvements in sentence completion accuracy were observed following stimulation of lobule VII but not lobule V. This behavioral specificity was paralleled by increased activation in left frontal and temporal cortices following posterolateral stimulation, suggesting functional coupling between right cerebellar posterior regions and classical cortical language areas. Together, task-based fMRI findings indicate that stimulation of the right posterolateral cerebellum can modulate cortical language-network activity, particularly during tasks involving contextual integration or prediction.

Two studies examined resting-state functional connectivity following cerebellar tDCS. Anodal stimulation of lobule VII altered intrinsic connectivity within language-related networks, increasing coupling between cerebellar regions and left-hemisphere cortical language areas. Specifically, enhanced interaction was observed between cerebellar regions associated with cognitive processing and cortical regions involved in speech motor control. These findings suggest that cerebellar stimulation can reshape large-scale network organization beyond task-specific contexts, influencing baseline connectivity within language circuits.

One study combined cerebellar TMS with EEG during reading tasks and a delayed recognition memory test. Using frequency-specific stimulation, the authors demonstrated dissociable effects on neural indices of semantic prediction and episodic memory encoding. Theta-frequency stimulation enhanced episodic encoding processes without affecting semantic prediction, whereas beta-frequency stimulation increased neural signals associated with semantic prediction but did not influence episodic encoding. This indicates that cerebellar contributions to language-related and memory processes may be frequency-dependent and mechanistically distinct, influencing predictive versus mnemonic processes through different oscillatory dynamics.

One clinical study combined repeated cerebellar tDCS with fNIRS in individuals with aphasia. Although both the active and sham groups showed language improvements following therapy, the active stimulation group demonstrated modulation of functional connectivity within left-hemisphere language networks. After baseline adjustment, the intervention group exhibited stronger network-level changes. This suggests that cerebellar stimulation may enhance reorganization of cortical language networks during recovery, even when behavioral differences are modest.

Overall, the neuroimaging and electrophysiological literature converges on the view that cerebellar stimulation modulates distributed language networks rather than exerting isolated local effects. These findings complement behavioral evidence by demonstrating that right posterolateral cerebellar regions are functionally integrated with cortical language systems and can influence their activation dynamics and connectivity patterns. Taken together, the reviewed literature indicates that cerebellar stimulation modulates language processing in selective and context-dependent manners, with effects most consistently observed following stimulation of the right posterolateral cerebellum. These findings raise important questions regarding the functional role of the cerebellum within distributed language networks, which are addressed in the following discussion.

## 4 Discussion

**Figure 4:**
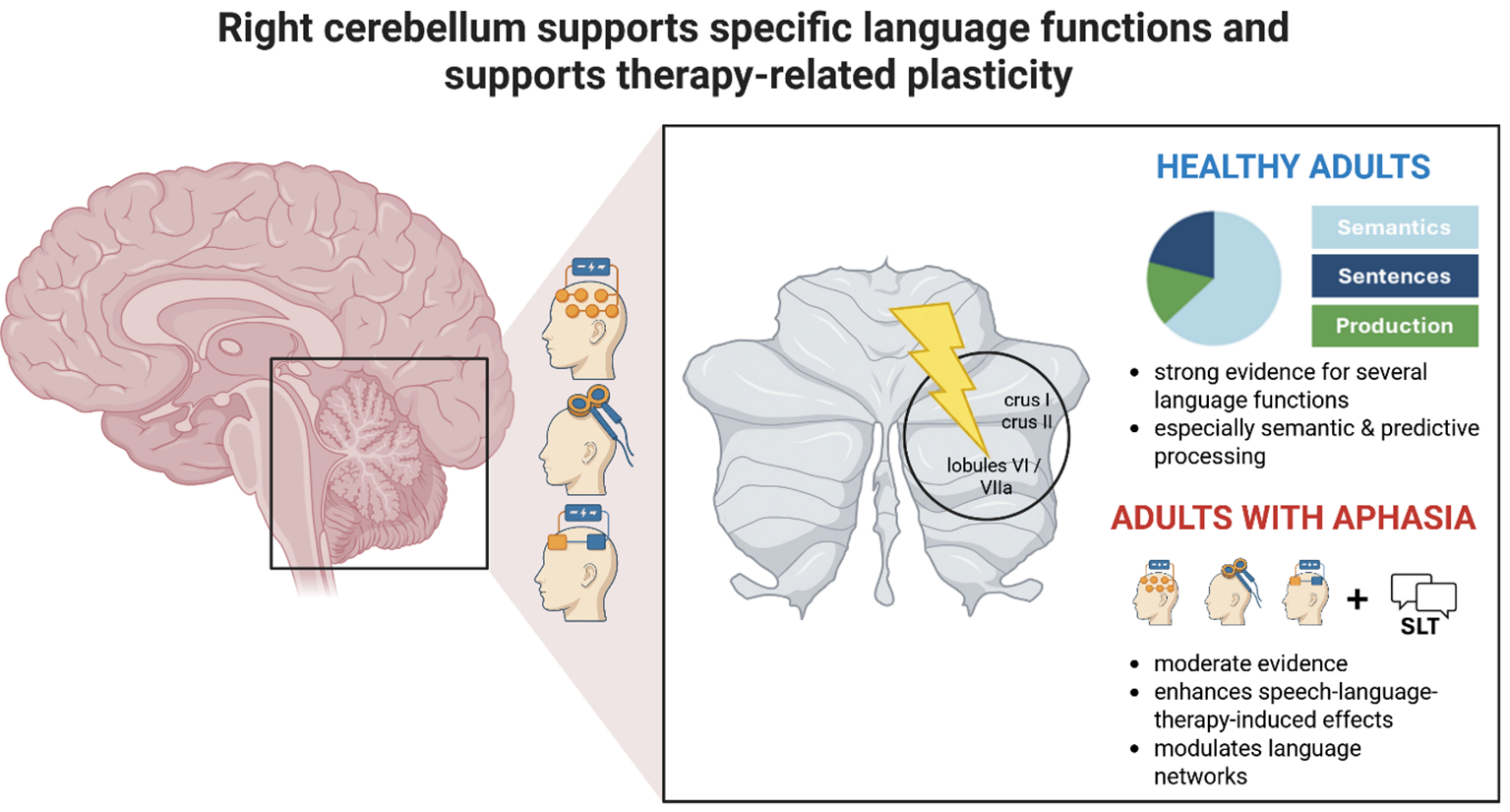
Schematic summary of results of the present review. Findings suggest that the right cerebellum is crucial for specific language functions, especially semantic prediction, speech production processes (naming, generation, reading) and sentence processing. In adults with aphasia, NIBS seems very promising in addition to SLT and has been shown to induce neural effects.

The findings of the present review indicate that cerebellar neurostimulation does not broadly enhance or disrupt language function in a uniform fashion. Rather, they suggest that the cerebellum contributes to language not by representing linguistic content, but by dynamically modulating predictive and adaptive processes within distributed language networks. Across different language and reading paradigms and healthy and clinical populations, stimulation effects were selective and most consistently observed following modulation of the right posterolateral cerebellum (see schematic illustration of results in **Figure 4**).

Stimulation effects seem to be most consistent under conditions that require some form of contextual integration, such as semantic or predictive processing. This pattern is consistent with theoretical accounts suggesting that the cerebellum is involved in domain-general sequencing and timing mechanisms (76, 77), which become highly relevant when linguistic processing requires the integration of temporally or structurally organized information. Most studies using semantic tasks used TMS, however, which has a higher precision and usually shows stronger effects than tDCS, which might influence the results. Nonetheless, instead of producing strong performance improvements or shifts in general processing, cerebellar stimulation typically influences highly specific contrasts or parameters within tasks. Importantly, this context sensitivity mirrors the task domains identified in the present review (semantic processing, sentence processing and comprehension, and speech production related tasks). Overall, the observation of context-dependent effects aligns with theoretical models proposing that the cerebellum is important for predictive processing through internal forward models (67, 78).

Across the included studies, several converging findings emerged: contrast-specific behavioral modulation, parameter-specific speech adaptation, and frequency-dependent neural dissociations observed in EEG studies. Together with evidence of altered cortico-cerebellar connectivity following stimulation, these findings are consistent with predictive and forward model accounts of cerebellar function (79). Within such frameworks, the cerebellum contributes not as a storage site for linguistic representations, but as a modulatory structure that refines and coordinates processing through anticipatory modeling and error-based updating. This emphasizes the role of the cerebellum in the coordination and fine-tuning of cognitive processes (14) and aligns with converging evidence from lesion and meta-analytic studies showing that cerebellar damage does not typically abolish linguistic representations, but instead leads to subtle impairments in tasks requiring controlled retrieval, sequencing, or integration (15, 17, 80). The present stimulation findings extend this literature by demonstrating that transient modulation of cerebellar activity produces similarly selective, context-dependent effects.

A network-level interpretation of the present findings may further help explain the functional specificity of cerebellar stimulation effects. The majority of included studies targeted right posterolateral cerebellar regions, particularly crus I/II and lobule VII as a more general target, which are known to show strong structural and functional connectivity with distributed cortical association networks, including inferior frontal, temporal, parietal, and frontoparietal control regions (8–10). Rather than acting as isolated linguistic processors, these cerebellar regions likely participate in large-scale cerebro-cerebellar circuits that support predictive integration, sequencing, executive control, and adaptive processing during language tasks. The observed task specificity across semantic prediction, sentence processing, verbal fluency, and speech adaptation paradigms may therefore reflect differential modulation of distributed cortical language systems rather than localized cerebellar computations alone. Importantly, the relative absence of effects in lower-level perceptual tasks, such as letter recognition or speech perception, aligns with the notion that posterolateral cerebellar regions are preferentially coupled to higher-order associative networks rather than primary sensory systems. Future studies combining individualized connectivity mapping, diffusion imaging, and network-informed stimulation approaches may help clarify how distinct cerebellar subregions contribute to specific language functions through dynamic interactions with cortical networks.

Importantly, the current body of stimulation research remains strongly right-lateralized in its methodological focus. The relative scarcity of left cerebellar stimulation studies therefore limits conclusions regarding hemispheric specialization. Rather than reflecting strict hemispheric specialization, however, the apparent right-hemisphere dominance in the included stimulation studies may arise from historical targeting conventions and assumptions about crossed cerebro-cerebellar connectivity. Recent work highlights the need to move beyond unilateral models and to investigate how bilateral cerebellar contributions interact within distributed language networks (15, 80). Future investigations should therefore incorporate direct hemispheric comparisons and network-level analyses to clarify whether functional asymmetries reflect true specialization or methodological bias.

With regard to clinical implications, single-session studies demonstrate that cerebellar modulation can influence language performance, but more consistent improvements appear in repeated-session protocols combined with therapy. This is in line with neurostimulation studies targeting learning disorders (75) or cortical targets for aphasia treatment (e.g., 81). However, even in these cases, behavioral effect sizes are often modest relative to the observed changes in neural processing (e.g., functional connectivity). This discrepancy raises important questions about the relationship between network reorganization and measurable behavioral gain. Given the cerebellum’s dense neuronal architecture and its integration within cortico-cerebellar loops, it is plausible that stimulation primarily reshapes network dynamics in ways that may require sustained training or task engagement to translate into stable behavioral improvements. Such dissociations between neural and behavioral effects are consistent with proposals that cerebellar contributions operate primarily through the optimization of internal models within cortico-cerebellar loops (82), where changes in network dynamics may precede measurable behavioral improvements or occur in the absence of strong behavioral effects. Although fewer studies directly targeted reading, the observed effects in sentence-level and predictive paradigms are consistent with prior accounts implicating the cerebellum in phonological decoding, automatization, and predictive processing during reading (42, 83, 84).

Taken together, the evidence positions cerebellar stimulation as a modulatory adjunct rather than a primary therapeutic target. From a mechanistic perspective, cerebellar stimulation likely influences linguistic processes by modulating the excitability of cerebello-cortical pathways and altering the efficiency of signal propagation within these loops (85–87), which may account for the often subtle and task-specific behavioral effects observed. At the same time, the literature is characterized by substantial heterogeneity in task paradigms, stimulation parameters, and outcome measures, often combined with small sample sizes and limited mechanistic biomarkers. These factors constrain direct comparisons across studies and limit immediate clinical translation. A more systematic integration of stimulation protocols with connectivity measures and computational models of cortico-cerebellar function may help clarify mechanisms of action and optimize intervention strategies.

Looking forward, advances in NIBS, such as state-dependent and network-informed protocols, individualized electric field modeling, multifocal stimulation approaches, and accelerated treatment schedules, hold considerable promise for improving target engagement and therapeutic precision. One potential avenue would be to combine cerebellar stimulation with cortical targeting to increase the efficiency of behavioral outcomes. In such paradigms, cerebellar stimulation may be prime cortical targets or help tailor stimulation to individual deficit profiles in patients with post-stroke language impairments. Realizing this potential will require deeper mechanistic insight into cerebello-cortical dynamics and the development of adaptive, biomarker-driven stimulation strategies that account for individual variability in network organization, task demands, and recovery trajectories.

These methodological limitations are particularly critical in the cerebellum, where anatomical depth, folding, and connectivity profiles differ substantially across lobules. As a result, inconsistent targeting and dosing may obscure functionally specific effects, contributing to variability across studies and potentially underestimating the cerebellum’s role in language. For instance, there is evidence that coil geometry and tissue depth influence the efficacy of stimulation in cerebellar regions (88). Most included studies did not use neuronavigation or systematically account for factors such as target depth and coil shape. This, together with variability in stimulation intensity, represents a significant limitation. Given the heterogeneous anatomy and depth of cerebellar subregions, such variability may obscure specific effects and contribute to inconsistencies across studies, particularly in higher-order cognitive domains. There is evidence that stimulation intensity varies across regions and is influenced by multiple parameters, highlighting the importance of dosing in future research (e.g., see 89, 90).

In sum, the available evidence supports a view of the cerebellum as a context-sensitive coordinator within distributed language systems, contributing to predictive integration and adaptive processing rather than serving as a primary linguistic substrate. Unlike prior consensus reviews that have broadly addressed cerebellar NIBS across motor and clinical applications, the present work provides a focused synthesis of language-related stimulation studies. By integrating findings across semantic, predictive, and speech-motor paradigms, this review highlights a consistent pattern of context-sensitive modulation within distributed language networks. Continued methodological refinement and theory-driven experiments will be essential to further delineate its role in language and harness its modulatory capacity for targeted and mechanism-informed clinical application. The current evidence suggests that repeated-session stimulation protocols targeting the right posterolateral cerebellum, particularly lobule VII/crus I–II, may hold promise as adjunctive interventions for post-stroke aphasia rehabilitation when combined with SLT. Future clinical progress will likely depend on individualized and connectivity-informed approaches that integrate structural and functional network mapping, optimize stimulation dosing and targeting precision, and account for inter-individual variability in lesion location, language impairment profiles, and recovery trajectories. More focal stimulation approaches, such as HD-tDCS or neuronavigated TMS protocols, combined with multimodal neuroimaging and longitudinal outcome assessments, may further help clarify which patients and language domains are most responsive to cerebellar neuromodulation.

### 4.1 Methodological limitations of the current evidence

Several methodological limitations of the current cerebellar neurostimulation literature should be considered when interpreting the reviewed findings. First, many studies included relatively small sample sizes, particularly in clinical populations. Across healthy participant studies, sample sizes typically ranged between 12 and 35 participants per condition, although several studies included fewer than 20 participants. Clinical studies were generally similarly small, with sample sizes often ranging between 6 and 25 participants. The smallest included study investigated six individuals with aphasia (50), whereas the largest healthy-participant study included 73 participants (63). Such sample sizes reduce statistical power and may limit the reproducibility and generalizability of reported effects, particularly given substantial inter-individual variability in responsiveness to NIBS.

Second, stimulation protocols and targeting procedures varied considerably across studies. Most included studies employed sham-controlled designs, especially studies using tDCS. TMS studies, on the other hand, primarily used a control site or no stimulation as control. We would also like to mention that reporting of blinding procedures varied substantially. While several studies explicitly described double-blind procedures, others provided only limited methodological detail regarding participant or experimenter blinding. This is particularly relevant for cerebellar stimulation paradigms, as these may induce perceptible sensory experiences that can compromise blinding efficacy and influence expectancy effects.

Third, targeting the cerebellar location varied substantially from study to study, making replications challenging and limiting conclusions regarding the specific cerebellar (sub-)regions. Only a minority of investigations used neuronavigation or MRI-/MNI-guided, precise targeting approaches, whereas most relied on scalp-based positioning relative to the inion. Moreover, scalp-based position relative to the inion also varied between studies targeting the same cerebellar area, highlighting how minor differences in positioning complicate comparisons between scalp-based targeting approaches. These limitations are particularly relevant in the cerebellum because anatomical depth, folding, and tissue geometry vary substantially across lobules, affecting the strength and distribution of induced electric fields. In TMS studies, coil geometry and target depth may further influence stimulation efficacy and reproducibility (88). In addition, most electrical stimulation studies employed conventional tDCS montages rather than more focal approaches such as HD-tDCS, likely resulting in stimulation of multiple cerebellar subregions rather than the specific target region defined by the experimental design or prior evidence. Consequently, stimulation specificity likely varied substantially across studies.

Fourth, replication across paradigms remains limited. Several reported effects stem from single studies or isolated datasets, and only a small number of paradigms, particularly semantic priming and sentence prediction task, have been investigated repeatedly across independent studies or laboratories. This limitation is especially important given broader concerns regarding reproducibility in cognitive neurostimulation research and calls for future replication studies to confirm the observed findings.

Fifth, relatively few studies combined cerebellar stimulation with neurophysiological or neuroimaging measures. Consequently, mechanistic interpretations regarding how cerebellar stimulation modulates distributed language networks remain constrained. Future studies would benefit from larger multi-session designs, standardized targeting approaches, improved reporting of blinding procedures, and multimodal methodologies integrating behavioral and neural outcome measures. 

In addition to the study-inherent limitations, one major limitation of the present systematic review concerns the screening procedure. Titles and abstracts were initially screened by one author and subsequently verified by a second author rather than using a fully independent dual-review process. Although disagreements were discussed and resolved collaboratively, this approach may increase the risk of selection bias or missed studies relative to fully independent screening procedures.

## Data Availability

The original contributions presented in the study are included in the article. The comprehensive study details, the R code to reproduce the figures and the data sheet for using the R code can be found on this OSF project page: 10.17605/OSF.IO/9C4PS. Further inquiries can be directed to the corresponding author.
